# Household disaster awareness and preparedness: A case study of flood hazards in Asamankese in the West Akim Municipality of Ghana

**DOI:** 10.4102/jamba.v11i1.789

**Published:** 2019-11-25

**Authors:** Frank J. Glago

**Affiliations:** 1 Akatsi College of Education, Akatsi, Akatsi South District, Ghana

**Keywords:** disaster risk awareness, disaster preparedness, Asamankese, flood-prone zone, West Akim Municipality

## Abstract

Increasing disasters and their associated devastating impacts on society have called into question the capacity of countries to address disaster occurrences. Hitherto, primary disaster management institutions have addressed disaster in a piecemeal manner, commonly through the distribution of relief items after occurrence of disasters. Considering this shortfall and as a contribution to the current discourse of disaster management, this study investigated households’ awareness and preparedness for flood disasters in Asamankese, a rapidly developing township, which has also seen increase in flood disasters in recent times. To this end, a mixed research method approach was used in both data collection and analysis. A survey was conducted to collect data from 200 households in the township. Two focus group discussions were also organised to gather in-depth insights. The study found that households’ awareness of flood disaster risks was very high in both flood-prone and non-flood-prone ecological zones of Asamankese. Also, notable from the study was that whereas level of awareness was high among residents, preparedness levels were generally low, especially in terms of financial preparedness. Several recommendations were proposed, which include improving public education and sensitisation on flood disaster preparedness strategies, creating financial support scheme for residents to increase their financial preparedness as well as encouraging residents to increase their social capital support and participate in community gatherings.

## Introduction

While disaster events in the past three decades have increased in frequency, their spatial distribution has made them a global phenomenon (Amoako & Boamah 2015). In addition, the impact of disasters has been quite devastating, claiming lots of lives daily (Oteng-Ababio 2013). Disasters can result from forces of nature, which may be aided by activities of man such as the construction of roads, irrigation and building of other infrastructure (Ogbanga 2015; Pokhrel 2015). Disasters may occur in the form of drought, fire outbreak, earthquake, tsunami, windstorm, flood, among others. What these events share in common is their ability to cause widespread community disruption, displacement, economic loss, property damage, deaths and injury as well as profound emotional suffering (Gillis, Shoup & Sicat 2001; Ogbanga 2015). According to the Internal Displacement Monitoring Centre (IDMC, 2015), disasters caused by natural hazards have displaced on average 26.4 million people annually between 2008 and 2015, which is equivalent to one person per second (International Federation of Red Cross and Red Crescent Societies [IFRC] 2016).

According to Fara (2001), there is no such thing as natural disaster. Events such as earthquakes, cyclones, tsunamis, volcanic eruptions, landslides, storms, fires, droughts and floods by themselves are not considered disasters. Rather, they become disasters when they adversely affect human life, livelihoods and property (IFRC 2007; Sinnakaudan et al. 2003; White 1945). While disaster events are not limited to a geographical space, their impact and the ability to recover from them varies significantly across space, with developing countries being the most affected areas (ActionAid 2006; World Bank 2010). Climate change, environmental degradation, population growth, increasing urbanisation, unsustainable development in hazard-prone environments, risky technologies, growing social and economic inequalities have all contributed to a dramatic increase in disaster events (Kötter 2003; Perrow 2007).

The persistent increase in the occurrence of disasters poses a substantive danger to the achievement of both sustainable development and poverty reduction initiatives (United Nations Office for Disaster Risk Reduction [UNISDR] 2009). The UNISDR (2009) defines disaster as follows:

[*A*] serious disruption of the functioning of a community or a society causing widespread human, material, economic or environmental losses which exceed the ability of the affected community or society to cope using its own resources. (https://www.eird.org/eng/terminologia-eng.htm). (n.p.)

The Oxford Reference Dictionary (ORD) defines flood as an overflowing or influx of water beyond its normal confines. Floods usually occur when the volume of water within a water body or water channel exceeds its carrying capacity, and as a result flows outside the normal perimeter of the water body (Adams 2008). Impacts of disasters may include loss of life, injury, disease and other negative effects on human, physical, mental and social well-being, together with damage to property, destruction of assets, loss of services, social and economic disruption and environmental degradation (UNISDR 2009).

Risk is usually associated with the human inability to cope with a particular situation. It comprises exposure to danger, adverse or undesirable prospects and conditions that contribute to danger (Hewett 1997). Sayers, Hall and Meadowcroft (2002) define risk as the probability of an event’s occurrence linked to its possible consequences. Disaster risks therefore denote the probability of disaster occurrence. Individuals, cities, and government, social and civil groups from various disciplines take into account the significance of sustained efforts to mitigate social, environmental, economic and emotional cost of disaster by addressing disaster risks (UNISDR 2002).

Among disaster events that have gained significant attention in recent times are those caused by floods. Flood disasters are vicious threats, rather than a natural occurrence when humans interfere with flood plains, and their management requires appropriate action at various scales and local community involvement (Anderson 1991; Douglas 2017). Although national and international institutions across the globe have developed and implemented programmes intended to control flood disasters, the phenomena persist (Bichard & Thurairajah 2014). On the global scale, flood disaster occurrences are phenomenal, and are probably the widest spread disasters that occur in most countries and cause maximum deaths (IFRC 2016). According to the United Nations Regional Coordinator in Dakar (October 2007), the worst flooding in 30 years, that battered West Africa in July 2007, caused more than 210 deaths and affected more than 785 000 people (Oppong 2011).

Disaster risk awareness, which denotes the extent of common knowledge about disaster risks, and the factors that lead to disasters, influence the actions that could be taken individually or collectively to address exposure and vulnerability to hazards. Awareness is a very crucial element for a society to effectively adapt to a flood risk. As stated by Shen (2009), awareness is diminished when the provision of an appropriate information is minimal or when memories of past experiences or events are diminished. Awareness can generally be uplifted through efforts that are centred on local issues, contain simple solutions to reduce flood risk and are repeated on regular basis (Poortinga, Bronstering & Lannon 2011).

United Nations Disaster Relief Organisation (UNDRO 1991) defines disaster preparedness as the state of taking direct and indirect measures to reduce damages that accompany disaster events to the minimum level possible. The objectives of preparedness are to ensure that appropriate mechanisms and resources are in place to assist those afflicted by the disaster and enable them to help themselves (United Nations Development Programme [UNDP] 1992).

Awareness and preparedness towards disasters vary depending on the characteristics of individuals within the community and characteristics of communities across space (Gerdan 2014; IFRC 2011). For instance, Gerdan (2014) has suggested that there is a direct link between education or sensitisation and awareness. Using educational levels of respondents, Gerdan (2014) found that higher levels of education contributed to producing positive awareness. In addition to this, the Regional Office for the Arab States of the United Nations Office for Disaster Risk Reduction (formerly UNISDR-ROAS) (USAID 2011) have indicated that depending on the type of community, access to information may vary depending on the social grouping and therefore one’s awareness of disaster risks. These groups may include gender, ethnic grouping and social status. Lastly, IFRC (2011) suggests that most people become disaster-aware based on their own personal experiences with disaster events over time.

The link between preparedness and awareness is well understood (Gerdan 2014; Sinclair & Pegram 2003), and as suggested by Gerdan (2014:159): ‘It is possible to increase the capacity to cope with the disasters, by raising the awareness of all components, all individuals and communities in line with this common cause’.

The aftermaths of flood disasters in Ghana are the large-scale destruction of infrastructure, displacement of people, loss of human lives, outbreak of diseases, huge loss of investments, among other things. Over the years, the government and disaster management agencies of Ghana have mainly focused on disaster relief activities after the occurrence of disasters (Oteng-Ababio 2013).

Ghana, similar to other African countries, has had a fair share of flood disasters in recent times, with urban areas having a disproportionate share of floods (Global Facility for Disaster Reduction and Recovery [GFDRR] 2014; Okyere, Yacouba & Gilgenbach 2012). By way of example, in 2007, a catastrophic flood in the northern region of Ghana affected more than 325 000 people, with approximately 100 000 people requiring assistance for the restoration of their livelihoods (GFDRR 2014). In addition, a more recent and perhaps the most devastating flood in the history of Ghana occurred in Accra on 03 June 2015 where 159 people lost their lives and several others were rendered homeless (Daily Graphic 2015). National Disaster Management Organisation (NADMO 2010) suggests that although Ghana is vulnerable to certain disasters, floods have been the major disaster that the country has faced in recent years, especially in urban areas (Kordie 2013).

The West Akim Municipality of Ghana is generally considered a flood-prone area. The municipality experiences serious perennial floods that cause loss of lives and destruction to properties. As a result, some residents of Asamankese township in the municipality abandon their homes at the slightest rainfall (Golden Gazette 2014). In early October of 2018, a mother and child died when their house carved in after five hours of continuous downpour that caused flooding in several communities of Asamankese township. Thousands of other residents in various communities of the municipality were also displaced (Ansah 2018). According to the West Akim municipal office of NADMO, 33 major flood events have been recorded in Asamankese township between 2009 and 2018, which means on average three major floods in the township annually. This resulted in distracting movements of residents, hindering pursuance of vital economic activities and rendering many residents homeless. Properties worth GH₵72 550.00 (about $20 000.00) were directly damaged during this period. In February 2015, for instance, torrential rains rendered some 50 families homeless in Asamankese, destroying properties worth thousands of cedis (Ghana News 2015).

In many countries such as the Netherlands, Germany, Italy, Japan and Bangladesh, extensive research has been done to access households’ preparedness for flood disasters (see Mallick et al. 2005; Motoyoshi 2006; Takao et al. 2004; Thieken et al. 2007). Takao et al. (2004) conducted a survey on residents’ awareness and preparedness to tackle floods in Nagoya City of Japan in 2002; the authors revealed that residents’ preparedness was not dependent on anticipation of floods, rather on ownership of home and amount of damage experienced during previous floods. Such insightfulness becomes relevant in attempts to comprehensively manage flood disasters.

However, limited research is done to proffer nuanced understanding of awareness and, more importantly, preparedness of individual households towards disaster prevention in Ghana. Studies of preparedness on disasters have disproportionately looked at institutional preparedness (e.g. see Oteng-Ababio 2013). Little focus is given to flood issues in small and medium towns and, more importantly, on the level of preparedness in these areas to confront floods. It is based on this understanding that the present research has tried to improve knowledge about the awareness and preparedness of individual households towards flood disaster risk prevention in Asamankese township. The research further interrogated some important factors that affect households’ level of awareness and preparedness to flood disasters in the study area. Level of awareness about flood disaster risk among residents was checked from knowledge of both physical and human-induced factors that contribute to floods in the area. In a similar fashion, level of individual households’ preparedness to flood disasters was also looked at from financial as well as social preparedness perspective. This study is therefore critical to understanding and empowering individual households on disaster management.

## Data and methods

### Profile of study area

Asamankese township is the capital town of West Akim Municipality. The topography of the municipality is generally mountainous and undulating. The municipality can be categorised as both lowland and highland area. The highest point is found around the Atiwa range, which is about 1250 ft. above sea level and is located between Pabi, Wawase and Asamankese in the northern part of the municipality (Ampadu-Agyei 2009). These conditions place Asamankese township in a valley-like landscape. A medium range, rising gradually between 500 ft and 1200 ft above sea level can be found in the eastern part of the municipality. The rest of the municipality is characterised by relatively lowland areas. The general elevation of Asamankese is about 500 ft. above the sea level (Ampadu-Agyei 2009; http://www.floodmap.net).

The West Akim Municipality falls within the semi-equatorial climatic zone. The municipality, similar to many parts of southern Ghana, is characterised by a double maxima rainfall regime and a period of dry spell (Ampadu-Agyei 2009). The mean annual rainfall is between 1238 mm and 1660 mm. Temperature is mostly high throughout the year, yielding an average of 26.1 °C (Ampadu-Agyei 2009).

Asamankese township is traversed by several streams which take their sources from highland areas and are seasonal in nature. The major rivers that traverse the township include Abukyen, Ayensu and Supon. These water bodies provide good opportunities of crop cultivation. Rivers and wells constitute the major sources of water, respectively providing approximately 33.1% and 35.1% of total water supply to Asamankese township (Karim et al. 2015).

**FIGURE 1:**
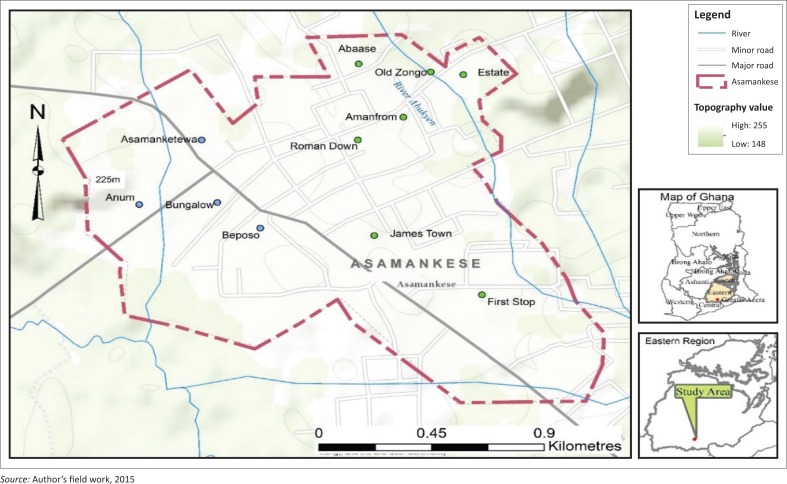
Map of Asamankese township.

Taking into consideration the topography and drainage as well as past experiences with flood events, the Asamankese township has been divided into flood-prone zone (hereafter referred as FPZ) situated on the eastern side to the township bordering river Abukyen, and non-flood-prone zone (hereafter referred as NFPZ) which covers mostly the western side of the municipality. The FPZ comprises the Old Zongo community, the Estate and the Abaase community (see Figure 1), whereas the NFPZ of the Asamankese township comprises the Anum, Asamanketewa and Beposo communities (see Figure 1).

With respect to population growth and physical expansion, Asamankese has been regarded as one of the fastest growing townships in the eastern region of Ghana (Ghana Statistical Service [GSS] 2010). The population of this township has increased from about 16 905 in 1970 to about 39 435 in 2010. Thus, within 40 years (from 1970 to 2010), Asamankese has recorded an increase of about 22 530 in population, thus representing about 133% increase between 1970 and 2010. This increase in population is partly attributed to the town’s strategic location and favourable soil conditions suitable for commercial agriculture, which has recorded an annual growth rate of about 2.5% (Danso-Wiredu 2011).

### Data

#### Sources of data collection

The primary quantitative data for this research were collected through a survey. The survey was aimed at soliciting the perceptions of residents on their level of awareness and preparedness towards flood disasters as well as factors that influence their awareness and preparedness levels. A five-item Likert scale ranging from ‘very high’ as the highest level of awareness to ‘very low’ being the lowest level of awareness was employed in the survey. Using this yardstick, as well as experiences from previous flood disasters, respondents were asked to rate their level of awareness of flood disaster risk. Two household heads were identified as key informants and were selected for both interviews and focus group discussions. Two focus group discussions were held with the residents, one with the residents from FPZ and another with the residents of NFPZ. This gave insights into the differences regarding the awareness and preparedness levels of the residents of different ecological zones of the municipality. Secondary data in the form of existing academic literature, magazines, print media and reports from various stakeholder institutions in disaster management were used to broaden the understanding of research area.

#### Sampling

The target population for the study was drawn from both FPZ and NFPZ ecological regions of Asamankese. The reason was to ascertain whether significant variations exist in the levels of awareness and preparedness towards flood disasters in different locations. Heads of households were purposely selected as points of contact from each household interviewed; they were selected because of their vital decision-making roles regarding their wellness and preparedness within the communities. These household heads mostly include men and women who have lived in their respective areas for more than two decades.

A total of 200 households from six communities in Asamankese township were selected to participate in the study. Using a stratified sampling method, the 200 households selected were divided into 120 households from FPZ (Old Zongo, Estate and Abaase communities) and 80 households from NFPZ (Anum, Asamanketewa and Beposo communities). This sampling was purported to highlight more issues of residents in FPZ and to be able to make substantial recommendations based on their responses to alleviate their challenges. Respondents sampled in various communities were proportional to the overall sample size of 200 respondents. Therein, respondents from Old Zongo (with a population of about 450 people) accounted for 24% of the entire 200 households visited. The Estate (with a population of about 300 people) and Abaase (with a population of about 250 people) communities respectively represented 21%, and 15% of the entire 200 respondents. In NFPZ, on the other hand, respondents from Anum (having a population of about 360 people) and Beposo (a population of about 350 people) represented 14% each of the entire selected households, whiles Asamanketewa (with a population of about 250 people) communities accounted for 12% of total households visited. After the strata were deduced, simple random sampling method was used to reach the required number of households in each of the communities. The choice of the simple random sampling at this stage was because population sizes of various communities were small, and such smaller communities had significant homogeneity within the population. Two five-member, mixed-gendered focus group discussions were held, which represented residents from both FPZ and NFPZ. The focus group members were selected among the households’ heads interviewed during the survey, so that one focus group discussion was held in each of the ecological zones with each member residing in the community for not less than 10 years.

### Ethical considerations

This article followed all ethical standards for a social science research and maintains the anonymity of its direct informants.

## Results and discussion

### Level of flood disaster awareness in Asamankese

**FIGURE 2:**
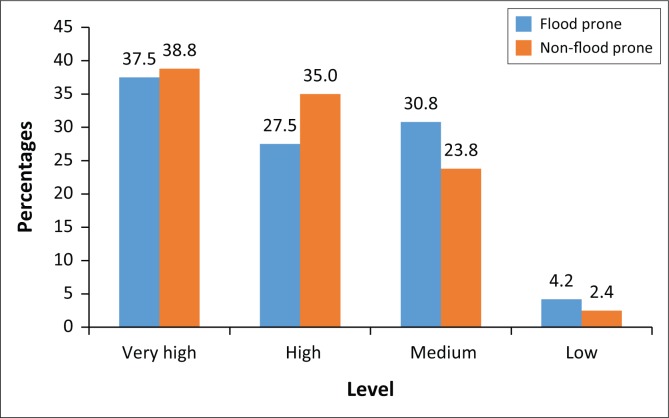
Level of awareness of flood disaster risks among residents of Asamankese.

Using the five-item Likert scale, the result, as shown in Figure 2, indicates that 37.5% of the respondents within FPZ recognised flood disaster as very high, 27.5% ranked it high and 30.8% recognised flood disaster as medium risk. The cumulative response for ‘very high and high’ gave an indication that indeed people were aware of floods as a serious disaster challenge, especially in FPZ. In the other ecological zone, respondents’ perception of flood disaster risk appeared to be equal as 38.8% of the respondents indicated flood disaster as very high. In addition, 35.0% and 23.8% of respondents, respectively, recognised high and medium levels of awareness to flood disaster risk. It is observed that there is an established high level of awareness to the flood disaster risks among residents of Asamankese. In addition, the findings show that residents’ high level of awareness to flood disaster risk is not dependent on the ecological zone in which they reside in Asamankese township.

### Means of flood disaster awareness

There are different modes through which communications regarding disaster risks are formed, disseminated and applied among various target groups (Ardaya, Evers & Ribbe 2017). Hence, this study sought to find sources through which residents of Asamankese were informed about flood disaster risks. In resonance with Shen (2009) and Takoa et al. (2004), which relate that memories of past experiences are central to shaping people’s awareness, the findings indicated that most people (about 61%) became aware of flood risks in Asamankese through their personal experiences with disaster events over time. As inferred from Table 1, majority of the respondents, about 68.3% and 51.2% from FPZ and NFPZ respectively, indicated that their awareness of flood disaster risk was the result of their personal experiences with flood events. Announcements on radio and occasional community meetings also accounted as other sources of flood disaster risks awareness within the two ecological zones.

**TABLE 1:** Source of flood disaster awareness among residents of Asamankese.

Source of awareness	Ecological zone				Total	
	Flood-prone zone		Non-flood-prone			
	*n*	%	*n*	%	*n*	%
Radio	21	17.5	14	17.5	35	17.5
Community meeting	11	9.2	12	15.0	23	11.5
Personal experience	82	68.3	41	51.2	123	61.5
Community labour	6	5.0	13	16.3	19	9.5

**Total**	**120**	**100.0**	**80**	**100.0**	**200**	**100**

### Respondents’ awareness of the effect of their physical environment and geography in flood disasters

Environmental factors that lead to disaster occurrences tend to increase one’s exposure and vulnerability to pending hazards. In view of this, this study sought to find out the level of respondents’ knowledge of the geography of the areas of their residence. When asked about why the area tends to experience much flooding, respondent in FPZ unsurprisingly asserted to the valley-like cosmos of the township, which makes it swampy. Proximity of settlements to river bodies that often overflow their banks was another issue raised. Statistically, 69.5% of the 120 respondents from FPZ alluded to the area being valley-like and hence swampy. It is therefore apparent that the residents of Asamankese do have fair knowledge of the areas’ natural vulnerability to flood.

### Resident’s awareness of human-induced factors contributing to flood disasters

Human-induced factors often heighten the exposure of communities in a natural flood-conducive environment to rampant flood disasters. This research therefore sought to investigate some human-induced factors that increase exposure of resident’s in Asamankese Township to frequent flood disasters. Factors highlighted from residents’ own perspectives are summarised in Table 2.

**TABLE 2:** Contribution of human-induced factors to flooding.

Human factors responsible for flooding in your settlement	Flood-prone area		Non-flood-prone area		Total	
	*n*	%	*n*	%	*n*	%
Building sited on water ways	19	15.8	21	26.3	40	20.0
Poor drainage system	47	39.2	32	40.0	79	39.5
Development of slums	44	36.7	17	21.3	61	30.5
Dumping of refuse in drains	4	3.3	2	2.4	6	3.0
Destruction of vegetated cover	6	5.0	8	10.0	14	7.0

**Total**	**120**	**100.0**	**80**	**100.0**	**200**	**100.0**

As seen in Table 2, significant proportion of residents in FPZ (39.2%) ranked poor drainage systems, 36.7% of respondents highlighted the development of slum and the activities from these areas such as improper disposal of waste, while 15.8% of respondents in FPZ also highlighted the building of houses on water ways as the human-induced factors heightening the exposure of the settlement to rampant flood disasters. Other factors raised include dumping of refuse in drains by some residents and destruction of vegetative cover which tends to enhance the free flow of surface water and reduces its rate of percolation, hence increasing floods’ susceptibility. These factors were equally shared by the NFPZ residents as being responsible for rampant floods occurring in the alternative ecological zone. On 17 October 2015, a 47-year-old head of a household residing in Anum, Asamankese township, recounted in a focus group discussion the following:

[*T*]he main problem in Old Zongo and Abaase areas is the gutters. The gutters are not enough to carry the water when it rains heavily, and secondly, they pour so much rubbish in the gutters, so some of the gutters are also full of rubbish. So, when it rains heavily, where will the water go, it must flood the area … the way we build in this area too is a problem. I even think government is not hard on people so we just build anyhow in the waterways. We in this area also experience floods but it is not serious like in Old Zongo areas, that is why we are always trying to tell people here not to build in the waterway, because of what is going on in Old Zongo and Abaase areas.

This narrative is not far from narratives related in previous literature. For instance, Karley (2009, cited in Amoako & Boamah 2015), related that:

[*A*]vailable evidence does not support the fact that, flooding in most parts of the country is as a result of unusual rainfall, rather, the problem results from the lack of drainage facilities to collect the storm water for safe disposal. (p. 25)

Braimah et al. (2014) also added that as many as 82% of their respondents indicated lack of drainage system, whereas 70% indicated improper disposal of waste or refuse.

### Individual level factors and concomitant level of flood disaster awareness

Setting aside physical geography, myriad other factors may also affect individual’s level of awareness of its environment (UNISDR-ROAS 2005). With that disposition, the study sought to analyse the relationship between individual level factors (that is, level of formal education, type of occupation and individual’s gender) and residents’ level of awareness of flood disaster risks. Consequently, a chi-square (*χ*^2^) test of independence was run at 95% confidence level (95% CI) (0.05) to ascertain the significance of these individual characteristics of their awareness levels.

Within FPZ, respondents with basic formal education and those with secondary education (39.4% and 33.3% respectively) constitute the majority of those who ranked flood disasters as the greatest disaster risks in their communities, compared to those with no formal education (15.2%) and tertiary education (12.1%) (see Table 3). Surprisingly, these two groups of residents (residents with basic formal education and secondary education) at the same time constitute the majority (20% of respondents with basic education and 60% with secondary school level education) of residents who claimed flood disasters were not a major risk in the township. Similar pattern was observed in the NFPZ of Asamankese township, where respondents with secondary school level education (53.6%) and tertiary level education (35.7%) significantly ranked flood disasters as a serious concern, but at the same time respondents with secondary school level education (38.7%) and tertiary level education (45.2%) ranked floods as not a serious disaster risk. Even though some variations were identified during cross tabulation, the chi-square test of association, in sync with Wang et al. (2018), showed no significant relationship existing between residents’ level of education and flood awareness in both ecological areas, as the *p*-values of 0.226 (FPZ) and 0.638 (NFPZ) were higher than the chosen 0.05 level of significance (see Table 3).

**TABLE 3:** Relationship between level of education and flood disaster awareness.

Individual level characteristics	Rating of flood awareness							
	Flood-prone zone				Non-flood-prone zone			
**Education**	1	2	3	4	1	2	3	4
None	15.2	16.2	0.0	6.7	3.6	10.5	0.0	3.2
Basic	39.4	27.0	20.0	20.0	7.1	10.5	50.0	12.9
Secondary	33.3	48.6	40.0	60.0	53.6	47.4	50.0	38.7
Tertiary	12.1	8.2	40.0	13.3	35.7	31.6	0.0	45.2

Note: Flood-prone zone – *c*^2^ statistic = 11.772, *df* = 9; *p* = 0.226 > 0.05. Non-flood-prone zone – *c*^2^ statistic = 6.897, *df* = 9; *p* = 0.648 > 0.05.

Regarding respondents’ occupation and their level of awareness of flood disaster risks in FPZ of Asamankese township, the private informal workers category had the highest level of awareness (63.7%) towards flood disaster risks (see Table 4). This supports the findings of Wang et al. (2018) on flood risk perception in Jingdezhen, China. Accordingly, self-employed residents (in this study as private informal workers) had the highest flood risk awareness reached using a range of 1–4 for the corresponding ‘very high’, ‘high’, ‘low’ and ‘very low’ levels of awareness. This suggests a more frequent experience with flood disasters among private informal workers, compared with formal government workers, private formal workers and farmers. However, as seen in Table 4, the private informal working class was represented significantly across various levels of perception on flood disaster risk in both ecological zones, compared with formal public servants, private formal workers and farmers. This skewed representation reflects the overall occupational constitution of residents in the township. Asamankese being the economic capital of West Akim municipality (Ministry of Finance and Economic Planning 2016), commerce is the major occupation of vast private informal traders and artisans and a minority population of formal public servants (Abdulai 2015). Private informal workers’ high awareness of flood disaster risks, as shown in Wang et al. (2018), might be explained by the fact that damages from flood disasters were solely borne by them at personal level, while private formal workers and public servants might receive some insurance cover from their place of work. The results of the chi-square test performed showed a significant relationship between occupation type and awareness of flood events in FPZ, given that the *p*-value obtained was less than 0.05 (0.047). Thus, residents’ occupation in FPZ somewhat influenced their level of flood disaster risk awareness in Asamankese.

**TABLE 4:** Relationship between individual’s type of occupation and rating of flood awareness.

Individual level characteristics	Rating of flood awareness							
	Flood-prone zone				Non-flood-prone zone			
**Occupation**	1	2	3	4	1	2	3	4
Government	12.1	0.0	20.0	2.1	10.7	15.8	50.0	12.9
Private informal	63.7	56.8	20.0	66.7	39.3	31.6	50.0	29.1
Private formal	12.1	8.1	20.0	15.6	10.7	10.5	0.0	41.9
Farming	12.1	35.1	40.0	15.6	39.3	42.1	0.0	16.1

Note: Flood-prone zone – *c*^2^ statistic=17.080, *df* = 9; *p* = 0.047 < 0.05. Non-flood-prone zone – *c*^2^ = 15.729, *df* = 9; *p* = 0.073 > 0.05.

The results of chi-square test performed for association in NFPZ, however, depict no significant relationship between residents’ occupation and flood awareness as *p* = 0.073 obtained is greater than the chosen level of significance. Hence, this indicates that generally one’s level of awareness of flood disaster risks, as concurred by Takao et al. (2004), is not dependent on one’s occupation.

The study again reached out to establish relationship between respondents’ gender and their rating of flood awareness using a range of 1–4 with the corresponding values of ‘very high’, ‘high’, ‘low’ and ‘very low’. The data indicated that a significant percentage of females were aware of floods within the study area. Specifically, a cross tabulation revealed that 63.6% and 64.3% of female respondents in FPZ AND NFPZ respectively were ranked very high for their flood disaster risk awareness as compared to 36.4% and 35.7% of male respondents in the corresponding zones as shown in Table 5. Although this high level of female awareness is reinforced by Wang et al. (2018), results of chi-square test about association indicated that there is no significant relationship between gender of respondents and their awareness of flood events in both zones as the respective *p*-values of 0.081 and 0.959 for FPZ and NFPZ were higher than the 0.05 level of significance.

**TABLE 5:** Relationship between individual’s gender and rating of flood awareness.

Individual level characteristics	Rating of flood awareness							
	Flood-prone zone				Non-flood-prone zone			
**Sex**	1	2	3	4	1	2	3	4
Male	36.4	51.4	80.0	62.2	35.7	42.1	50.0	38.7
Female	63.6	48.6	20.0	37.8	64.3	57.9	50.0	61.3

Note: Flood-prone zone – *c*^2^ statistic = 6.687, *df* = 3; *p* = 0.081 > 0.05. Non-flood-prone zone – *c*^2^ statistic = 0.306, *df* = 3; *p* = 0.959 > 0.05.

In sum, with reference to the data obtained from the West Akim Municipal Office of the NADMO, it is evident that flood is a common disaster in Asamankese township. Again, it is also revealing that, aside residents’ occupation and level of awareness in FPZ, individual dynamic factors such as level of education, gender and occupation to a great extent, had no significant influence on the level of awareness of flood disaster risks, and thus, the latter, in congruence with Takao et al. (2004), was informed primarily by residents’ personal experiences with past flood events.

### Preparedness strategies adopted by households to confront flood disasters

#### Financial preparedness and resilience

As argued by Cannon (1994), awareness of flood disaster risks and one’s vulnerabilities are insufficient in reducing their impacts, lest it is coupled with an understanding of different economic systems and economic capacities of people to withstand and recover from disasters. The study therefore sought to find out how prepared were residents in terms of their economic resilience. For this, the respondents were asked their assured means of sustenance (if any at all) should they become victims of flood disaster. The results summarised in Table 6 show that of the 120 respondents in FPZ, only 28.3% had some means of sustaining themselves, while 71.7% indicated that they had no guaranteed means of sustenance should they become victims of floods. Similarly, majority of respondents (about 71.2%) in NFPZ had no assured means of sustenance in case of becoming flood victims. Only about 28.8% of respondents (see Table 6) indicated that they had an assured means of sustenance.

**TABLE 6:** Economic and social resilience to floods in Asamankese township.

Economic and social resilience	Flood-prone area		Non-flood-prone area		Total	
	*n*	%	*n*	%	*n*	%
**Do you have any means of sustenance**						
Yes	34	28.3	23	28.8	57	28.5
No	86	71.7	57	71.2	143	71.5
Total	120	100.0	80	100.0	200	100.0
**Which means**						
Savings	31	91.2	18	78.3	49	86.0
Insurance	3	8.8	5	21.7	8	14.0
Total	34	100	23	100	57	100
**Means of regaining property**						
Savings	43	35.8	20	25.0	63	31.5
Insurance property	4	3.3	1	1.2	5	2.5
Family support	68	56.7	51	63.8	119	59.5
NGO	5	4.2	8	10.0	13	6.5
Total	120	100.0	80	100.0	200	100.0

The result brings to the fore the issue pointed out by Flooding Issues Advisory Forum (FIAC, 2007) that sustainable flood management involves both social and economic resilience. Moreover, it also suggested that sensitisation should not only include communication of hazards or announcing impending floods but also education on the issues such as better economic planning within a catchment area.

In the case of those who had some means of sustenance, they were further inquired about the specific means of sustenance in anticipation of flood disasters. As summarised in Table 6, out of the 34 respondents in FPZ, 91.2% indicated that they had financial savings (referred to as *Susu* in Ghanaian parlance) as a recovery plan, while only 8.8% indicating that they rely on insurance. On the other hand, out of 23 respondents in NFPZ, 78.3% indicated that they had financial savings, while 21.7% indicated that they depend on insurance. The results thus show that a large proportion of respondents (about 71.7% and 71.2% in FPZ and NFPZ respectively) had no means of sustaining themselves to face flood disasters. For those who had some means of sustenance, they largely depended on their financial savings, and this was the situation across both ecological zones.

According to Grothmann and Reusswig (2004), self-protective behaviours by residents of flood-prone urban areas could help to scale down flood damage monetarily by 80%, which thus reduces the need for public flood risk management. Hence, this research further probed into the sufficiency of financial savings for the full recovery of residents from flood catastrophes. During the focus group discussions in FPZ, participants opined that their financial savings were often inadequate, and thus unlike Grothmann and Reusswig’s (2004) claim, couldn’t be sufficient to resuscitate them from flood catastrophes. A 62-year-old former teacher who participated in the focus group discussion held on 03 October 2015 in Old Zongo related as follows:

Some of the people here can do Susu, a lot of people too cannot do it because they don’t have the money. Even many people here pay their children’s school fees, they go to hospital when they are sick, and it is this same small ‘Susu’ that they take to do these things. So, when serious floods happen, and affects them, how much more will they have to cater for themselves from the small susu. They can buy food for some few months then the money finish. If their families too cannot contribute much, then even the small children may have to go to the market side to get something to do so they can survive.

This insufficiency of personal financial protection in times of flood disasters owes to low incomes earned by majority of the residents who were private informal workers (see Table 4), particularly in FPZ. Respondents were then asked how they would regain some basic possessions they lost in floods. The results presented in Table 6 show that just 35.8% and 25.0% of respondents in FPZ and NFPZ respectively could regain their possessions through savings. A larger number of respondents indicated that they would be able to regain their lost possessions in floods through the extended family social support. The results, therefore, suggest that although individual financial savings are crucial in meeting the very basic needs of food and clothing immediately after floods, the extended family support is essential to regain some lost possessions. In this regard, this study suggests that the focus should be on preparing of both areas of personal savings and insurance schemes for building resolute capacity to recover from flood disasters.

#### Social preparedness and resilience

**FIGURE 3:**
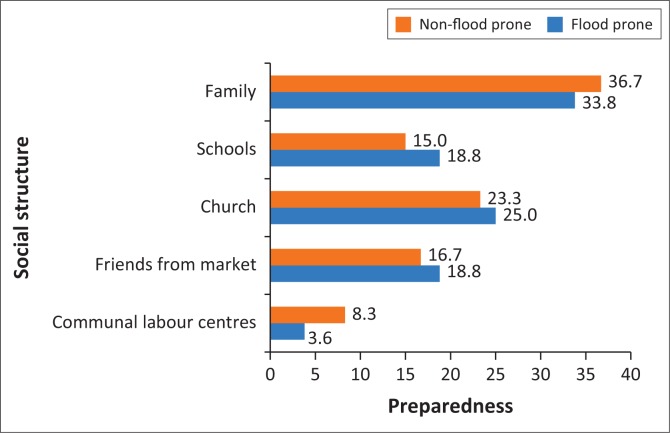
Social structures that enhance preparedness.

Continuing with the factors that are critical to enhance resilience, respondents were asked about the social structures that enhance their preparedness or serve as a conduit to improve their preparedness for floods. As presented in Figure 3, 36.7% and 33.8% of respondents in FPZ and NFPZ indicated that the family social structure was critical to prepare for floods. The result again shows that about a quarter (23.3% and 25.0% respectively) of respondents in FPZ and NFPZ asserted that the church was crucial to their preparedness for floods. This is because occasional announcements are made in the church regarding anticipated rainfall. The church then organises some cleanup exercises to help de-silt some choked gutters, especially around the church premises. Attending members then replicate the cleanup exercise in their homes in anticipation of heavy rains.

An equally important area noted was friends from the market. It is observed in Figure 3 that 16.7% of respondents from FPZ and 18.8% from NFPZ highlighted the importance of information from friends from market centres. Hence, it is imperative to realise the importance and significance of social capital and social institutions such as friends and family towards resilience-building and disaster preparedness of communities. It therefore goes without saying that in the adoption of any sustainable flood management strategy, social structures (such as families and community commercial activity centres) should be seriously considered. Therefore, it is important that churches and market centres should become places where information on imminent flood disasters from reliable sources be communicated. In addition, by improving individual economic resilience, the study shows that it would invariably strengthen not just one’s immediate nuclear family but also improve the preparedness and ability of other members of the extended family to recover from disasters.

## Conclusion

Flood disasters are major environmental challenges faced by residents of Asamankese, the capital of the West Akim Municipality in Ghana. Residents’ level of awareness of flood disaster risks tends to be high because of their own experiences irrespective of their individual level of education, occupation and gender. The rather rampant flood disasters in Asamankese are the combined result of natural environment and inappropriate human behaviours. Despite the high level of awareness of flood disaster risks, there appears to be an incommensurate level of flood disaster preparedness in the settlement. This is mainly because of low level of individual economic capacity to withstand and recover from these disasters.

In times of devastating floods, it is equally important to note the influence of strong social capital in one’s preparedness and ability to recover from flood disasters in the township. This strong element of social capital and institutions was the major means by which many people could recover their possessions that were lost in floods. Moreover, while an early warning system might be in place (partly through church gatherings and market centres), the fact is that preparedness for flood disasters in Asamankese is low, which means that people might not be able to respond properly to and recover fully from the impact of floods.

## Recommendations

The following recommendations are made from the findings of this study for promoting local and institutional reforms for awareness and preparedness towards flood disasters.

- Relevant institutions, such as private radio stations, in Asamankese should collaborate with state institutions to further organise these communities, educate them and support them to adopt appropriate adaptive skill-building techniques such as building on stilts and adherence to appropriate building codes. This is important because some of these institutions are already serving as sources of awareness of imminent floods to some sections of Asamankese residents.
- In addition, relevant state institutions should collaborate with social institutions such as churches in Asamankese to set up financial support schemes for residents. These schemes should collect financial contributions from local residents, institutions as well as government’s financial support. Such schemes if managed properly by local residents and existing institutions could become a reliable financial support to assist victims of floods in the township.
- Community-initiated mitigation measures, such as construction of new drains, de-silting of existing drains as well as expanding individual’s social capital by participating in social gatherings, should be vehemently encouraged by local community members to build a more socially resilient community.
